# High-density surface electromyography maps after computer-aided training in individual with congenital transverse deficiency: a case study

**DOI:** 10.1186/s12891-020-03694-4

**Published:** 2020-10-15

**Authors:** Katarzyna Kisiel-Sajewicz, Jarosław Marusiak, Mónica Rojas-Martínez, Damian Janecki, Sławomir Chomiak, Łukasz Kamiński, Joanna Mencel, Miguel Ángel Mañanas, Artur Jaskólski, Anna Jaskólska

**Affiliations:** 1 Department of Kinesiology, Faculty of Physiotherapy, University School of Physical Education in Wrocław, Al.I.J. Paderewskiego 35, P4, 51-612 Wrocław, Poland; 2 Department of Bioengineering, Faculty of Engineering, Universidad El Bosque, No 131 A, Ak. 9 #131a2, Bogotá, Colombia; 3 Biomedical Engineering Research Centre and Biomedical Research Networking Center in Bioengineering, Biomaterials and Nanomedicine, Universitat Politècnica de Catalunya, Avinguda Diagonal, 647, 08028 Barcelona, Spain

**Keywords:** Mental training, Virtual reality system, Congenital upper limb amputation, Trapezius muscle, Case report

## Abstract

**Background:**

The aim of this study was to determine whether computer-aided training (CAT) of motor tasks would increase muscle activity and change its spatial distribution in a patient with a bilateral upper-limb congenital transverse deficiency. We believe that our study makes a significant contribution to the literature because it demonstrates the usefulness of CAT in promoting the neuromuscular adaptation in people with congenital limb deficiencies and altered body image.

**Case presentation:**

The patient with bilateral upper-limb congenital transverse deficiency and the healthy control subject performed 12 weeks of the CAT. The subject’s task was to imagine reaching and grasping a book with the hand. Subjects were provided a visual animation of that movement and sensory feedback to facilitate the mental engagement to accomplish the task. High-density electromyography (HD-EMG; 64-electrode) were collected from the trapezius muscle during a shrug isometric contraction before and after 4, 8, 12 weeks of the training. After training, we observed in our patient changes in the spatial distribution of the activation, and the increased average intensity of the EMG maps and maximal force.

**Conclusions:**

These results, although from only one patient, suggest that mental training supported by computer-generated visual and sensory stimuli leads to beneficial changes in muscle strength and activity. The increased muscle activation and changed spatial distribution of the EMG activity after mental training may indicate the training-induced functional plasticity of the motor activation strategy within the trapezius muscle in individual with bilateral upper-limb congenital transverse deficiency. Marked changes in spatial distribution during the submaximal contraction in the patient after training could be associated with changes of the neural drive to the muscle, which corresponds with specific (unfamiliar for patient) motor task.

These findings are relevant to neuromuscular functional rehabilitation in patients with a bilateral upper-limb congenital transverse deficiency especially before and after upper limb transplantation and to development of the EMG based prostheses.

## Background

This study explored a powerful mental training method that may be administered before and after upper-extremity transplantation to induce plasticity of the neuromuscular system in humans with congenital absence of the upper limbs. We believe that engaging this population in computer-aided training (CAT) consisting of virtual reality and sensory feedback before and after upper-extremity transplantation would facilitate functional reorganization of the skeletal muscles and promote motor function recovery. Cases of bilateral upper-limb congenital transverse deficiency are sparse and we failed to find information in the literature about muscle activity and the effect of the mental training on reach and grasp skills as well as motor control plasticity in affected humans. On the other hand, consistent reorganization and modulation of the cortical sensorimotor map after limb amputation [1, 2] and bilateral hand transplants [3, 4] as well as behavior and skill acquisition [5–7] occur in response to activity as the central nervous system (CNS) can demonstrate substantial plasticity [5]. Expansion within the primary sensorimotor cortices of the cortical surface corresponding to unaffected body parts toward the adjacent de-afferented and de-efferented areas (i.e., corresponding to the missing limb) is now well established (e.g. [8, 9]).

Although it is unknown whether a person born without an upper extremity is able to develop the similar response as upper-extremity amputees [1–4], we suppose that lack of the upper limb affects trapezius muscle function as it never had to play a role in limb weight-bearing action and movement initiation. Based on the brain reorganization due to activity in primates [10, 11] and following limb loss (and a lack of sensory input), we expected to observe different effect of the CAT on the muscle activity in a congenitally amputated subject.

In patients with congenital amputation, CAT seems to be an appropriate intervention to induce and record potential changes in muscle activity (due to plastic changes in the CNS) during the learning process as the subject can move and observe the movement on the screen (visual feedback). The monitor shows the virtual upper limb facing away from the patient into the virtual world, giving the subject the impression that it is an extension of his own limbs. It has been shown that mental exercises improve motor performance during skill acquisition [12], in the recovery of motor function following a stroke [13], as well as to reduce phantom limb pain in patients after limb amputation [14]. Our study aims to show that the CAT can be applied in individuals with congenital absence of the upper limbs before the upper limb transplantation.

We assumed that the repetitive mental task supplemented by a visual task to move the joints (during CAT) activates brain-to-brain functional binding between the primary visual cortex (V1), extrastriate visual cortical areas (V2, V3, V4, and V5), and prefrontal cortex for more prompt recovery related to CNS re-organization (and may include establishing new synapses, “silent” synapse awakening, and neural networks for the control of upper-extremity movement) and will result in muscle activity adaptation. Electromyography (EMG) is a method that is used to detect muscle activity and indirectly reflects functional changes in the CNS during motor tasks [15]. Conventional EMG is utilized in myoelectric prostheses, allowing for the detection of myoelectric signals from only one or two electrode sites [16]. High-density surface EMG (HD-EMG) is a technique that uses closely spaced two-dimensional (2D) multielectrode arrays located on the surface of the skin [15, 17]. This multichannel electrode approach allows the recording of different temporal and spatial signal EMG amplitudes from the muscle to provide an HD-EMG map that could present activation patterns from different muscle parts [18] or determine the topography of the innervation zones (IZ) [19].

We assumed that CAT of a reaching and grasp movement would induce changes in HD-EMG map signal intensity and their spatial distribution of the trapezius muscle in a person with bilateral upper-limb congenital transverse deficiency. Therefore, the first purpose of this study was to assess whether and when changes in HD-EMG maps of the trapezius muscle result from CAT. The second purpose was to compare the HD-EMG maps between a healthy control person and a person with bilateral upper-limb congenital transverse deficiency after 4, 8 and 12-week training program.

We used a case-control design because this deficiency is very rare and no effective intervention has been reported to support a surgical treatment and recovery of the motor function before and after both hand transplantation. Although it will be impossible to generalize our results, they will be novel and significantly contribute to our understanding of the mechanisms of neuroplasticity due to congenital limb deficiency individuals after motor imagery training before transplantation. By strengthening our knowledge of the effectiveness of the CAT in congenitally amputated individuals before transplantation of the upper limbs, we may support a success of a surgical treatment and recovery of the motor function after transplantation. The proposed methodology of the CAT training and the method for the assessment of the efficiency of the training using HD-EMG maps in the patient could improve also the control of EMG based prostheses.

## Case presentation

One female patient with bilateral upper-limb congenital transverse deficiency (unknown origin) and one healthy female control participated in the present study. The 30-year-old patient, who had no upper extremities to the level of the glenohumeral joint, had a body mass of 45 kg, height of 150 cm, and body mass index of 19.8 and exhibited right dominance. The 27-year-old control had a body mass of 56 kg, height of 168 cm, and body mass index of 20. She exhibited a right dominance as evaluated with the Edinburgh Handedness Inventory. The patient used her feet and mouth for the hand related motor task as reaching and grasping. She had no history of phantom limb pain or neurological disorder (screening for neurological condition with head MRI).

The research reported in the paper was conducted in accordance with the Code of Ethics of the World Medical Association (Declaration of Helsinki) for experiments involving humans. The study was approved by the Review Board of the University School of Physical Education in Wroclaw. All subjects provided written informed consent before participating. This study is a part of registered experiment (ID: NCT04048083, clinicaltrials.gov, U.S. National Library of Medicine, Registered – 07.08.2019 Retrospectively registered).

### Experimental methodology

The HD-EMG, force measurements, and ultrasonographic (USG) screening results were evaluated before CAT (1 week before training) and at 4, 8, and 12 weeks of the training period. The control subject and patient were instructed not to exercise outside the laboratory during the 12-week training period. We chose a 12-week training program based on the knowledge that within this period we can induce long-term functional changes in the neuromuscular system [20].

### Instrumentation

The USG scanning was done using an Ultrasound Scanning System Echo Blaster 64/128 and a 9-cm-long linear transducer HL9.0/60/128Z (scanning frequency, 9 MHz) using Echo Wav II Version 1.6.0 software (Telemed Company, Vilnius, Lithuania). The force signal was measured using an Interface SML-1000 load cell force transducer (signal amplified by an interface strain gage amplifier) with a sampling rate of 2048 Hz. The high-density monopolar EMG signals were recorded by an EMG-USB2 64-channel device (OT Bioelettronica, Torino, Italy) with a sampling frequency of 2048 Hz, 3-dB bandwidth of 10–900 Hz, and 2000 programmable gains.

### USG recording

The USG screening was performed before the HD-EMG data collection sessions as a starting procedure. Scanning head was placed on the skin over the same area of the trapezius muscle at which the HD-EMG grid was placed bilaterally in both individuals. The ultrasound transducer was coated witch a transmission gel to provide acoustic conduction without depressing the dermal surface. The USG image was always made in longitudinal projection. The recorded area was located 1.5 cm below the virtual line between processus spinosus C7 and the point on the acromion at the acromioclavicular joint. The ultrasound transducer was centered on the described virtual line corresponding to the seventh electrode spot on the second row of the HD-EMG grid.

### Force recording

The force signal during a shrug isometric contraction was recorded with each subject in a sitting position. The force transducer was located on the cranial surface of the acromion vertically in line with the generated force vector. The subject’s task was to generate the maximal voluntary contraction (MVC) as quickly as possible; a bar representing the actual force value was displayed on the screen. The MVC measurement was taken three times and the computer calculated the 20% MVC value based on average of the three MVC values.

A bar representing 20% MVC was displayed on the computer screen and the subject was asked to reach and sustain this force level with visual feedback. The subjects performed five 10-s submaximal contractions with a 10-s rest between them.

### HD-EMG recording

The HD-EMG measurement was performed at rest and during maximal voluntary contraction (MVC) and submaximal (20% MVC) force. Given the different sizes of the shoulder segments in the subjects, 5 × 13 grid electrodes (OTbioLab ELSCHO064LS) were used in the control subject, whereas only 5 × 9 electrodes were used for the patient. The inter-electrode distance was 8 mm in both cases. The electrode array was positioned on the medial trapezius muscle [21].

The HD-EMG grid location was kept consistent during each session. To this end, the USG screening was performed to ensure that the electrode location was parallel to the high echogenicity lines representing fascial layers in the muscle belly. To increase the inter-test reliability factor of the grid position, a single manual therapist with long-standing experience was responsible for performing the USG screening, determining palpable landmarks, and locating the grid on the subject’s body during all sessions. A photo of the trapezius muscle with the grid located on it was also taken for further comparison.

The corner without an electrode was always proximal to the vertebra. The vertical axis location was as follows: the upper electrode grid row was located on a virtual line between processus spinosus C7 and the distal edge of the acromion at the acromioclavicular joint. The horizontal axis placement was as follows: the seventh column counted from the empty corner was in the middle of the line between the acromion and C7. According to Johnson et al. [22], this location covers the trapezius muscle fibers with transverse orientation that draw the clavicle, acromion, and scapula backward and medially. These movements contribute to the transition of the upper limb from the grasp back to the starting position.

The reference electrode (driven right leg, drl in and patient reference) were placed at the malleolus of the lower extremity contralateral to the recorded side (because of the congenital upper-extremity amputation of the examined individual) as recommended by the manufacturer.

#### Computer-aided training (CAT)

**Fig. 1:**
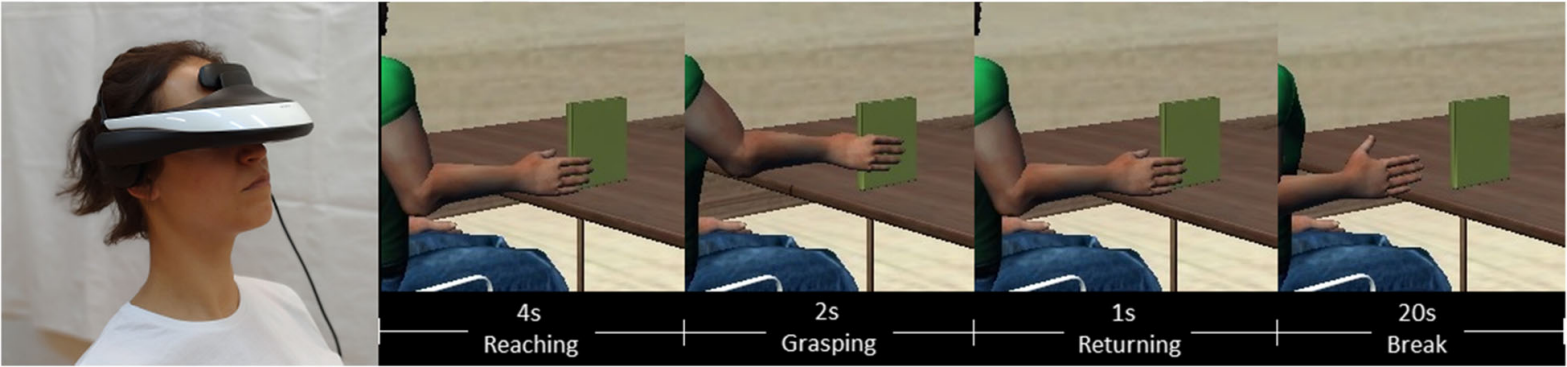
Example of reaching to grasp movement generated by the visualization software of the CAT system

CAT is a mental training program supplemented by visual and sensory tasks (Fig. 1). The mental exercise (motor imagery) was the mental execution of the reach task without any movement or muscle activation [23]. According to Ranganathan et al. [24], the mental process involved visualization-guided brain activation training. Our subjects were instructed not only to visualize themselves performing the reach task but to adopt a kinesthetic imagery approach in which they urged the muscles to contract because such training can be accompanied by a significant physiological response [24] and thus can potentially induce changes in the brain and skeletal muscles.

Both the patient and the healthy control subject underwent CAT of the reaching to grasp task in which their role was to visualize the movement of reaching to grasp a book using virtual reality goggles. The goggles (HMZ-T1; Sony, Tokyo, Japan) were controlled by visualization software coded specifically for this examination, we refer to [25] for more details. Each subject sitting on a chair wearing goggle had to perform simple tasks of reaching and precision fine grasping of a book using four fingers of the virtual upper extremity using the CAT system [25]. The vibrating devices to stimulate the mechanoreceptors were attached to the trapezius muscle in both subjects. An additional device was attached to the first dorsal interosseous muscle in the healthy control subject. The vibration were adjusted to each individual subject’s sensation to avoid any activation of the muscle (measured by surface EMG) through the primary muscle spindle afferent fibers stimulation. In this study during the training we did not observed muscle activation (monitored by EMG to distinguish motor imagery from aberrant motor execution of the reaching-to-grasp). All training sessions were documented in writing and recorded in the CAT system. The training was conducted by the same instructor sufficiently knowledgeable in the CAT system.

#### Training procedure

There were tree training sessions per week within 12 weeks. Each training session consisted of 30 trials of the reaching to grasp task (three sets of 10 trials each) for each upper limb. There was a 15-min rest between left and right limb. There was a 20-s rest between trials and 3-min rest between sets. A sample repetition is shown in Fig. 1.

Before each training session the subject with congenital transverse deficiency was asked to reach and pick up a real book using the leg (she usually does), whereas the healthy control subject was asked to use the hand. Each subject grasped and touched the book to feel its weight, size and surface texture. The sensory information are essential for generation of the reach-to-grasp motor command. The instruction was then read to each subject while she attempted to mentally reach and precisely grasp the virtual book. Each subject performed three practice trials after receiving the instructions. Subsequently, the instruction was discontinued and the subjects performed 30 mental movements by following auditory cues.

#### EMG analysis and statistics

The force signal (auxiliary inputs in the EMG signal) was used as the reference. The rising and falling edges of that signal were automatically detected and a signal segment before and after 1 s of those edges was taken for map calculation (to avoid muscle contraction onset and offset). The root mean square (RMS) was calculated on 250-ms epochs and averaged over all epochs for each channel (i.e. pixels in the image).

The signals were zero-phase filtered between 15 and 350 Hz with a 4th-order Butterworth filter after DC offset removal. Power line interference was reduced using an adaptive filter described elsewhere [26]. Activation maps were then obtained from the RMS of the individual channels as:

1 $$ {I}_{x,y}=\left(\frac{\sum \limits_{k=1}^M{RMS}_k\left({s}_{x,y}\right)}{M}\right) $$

where *s*_*x,y*_ was the EMG monopolar channel located at the position (*x, y)* of the 2D array and the RMS value was calculated in M non-overlapping 250-ms epochs on a segment where the force was constantly maintained at the target level within a threshold of ±2.5% MVC.

To describe changes in the activation maps between trials of the same session, two variables were calculated: 1) the 2D cross-correlation (*CC*) coefficient; and 2) the variance (*Var*) of the average intensity of the maps in each trial calculated as:

2 $$ {i}_{av}=\frac{\sum_x{\sum}_y{I}_{x,y}}{N} $$

where N is the total number of channels in the recording and *I, x* and *y* are as previously described.

On the other hand, changes between sessions were quantified using average intensity between trials (*i*_*av*_ - Eq. 2) and the center of gravity (*CoG*) of the maps (Eq. 3):

3 $$ CoG\left(x,y\right)=\frac{1}{\sum_x{\sum}_y{I}_{x,y}}\sum \limits_{x,y}{I}_{x,y}\cdot \left[\begin{array}{c}x\\ {}y\end{array}\right] $$

#### Innervation zone maps

The IZ of the trapezius muscle were determined during isometric submaximal contractions performed at 20% MVC. The IZ were automatically localized by an algorithm implemented by OTBiolab. The algorithm is based on the calculation of the phase inversion of the differential EMG signals. This plugin works with monopolar signals acquired by 64-channel matrixes. The phase inversion is found using a cross-correlation of the EMG signals from adjacent channels of the array (i.e., Channel 1 versus Channel 2, Channel 2 versus Channel 3, …, Channel 14 versus Channel 15). The cross-correlation was based on the following formula:

4 $$ {R}_{x,y}\left(\uptau \right)=\frac{\left(1/\mathrm{N}\right){\sum}_{i=1}^N\left({x}_i-\overline{x}\right)\left({\mathrm{y}}_{i+\tau \cdot fs}-\overline{y}\right)}{\left(1/\mathrm{N}\right)\sqrt{\sum_{i=1}^N{\left({x}_i-\overline{x}\right)}^2}\sqrt{\sum_{i=1}^N{\left(y-\overline{y}\right)}^2}} $$

where *Rx,y*(*τ*) is the cross-correlation function, *N* is the number of data points in the input signals, *τ* is the temporal phase shift between the two signals, and *fs* is the sampling rate. For each performed cross-correlation, the maximum cross-correlation coefficient was normalized between 0 and + 1 [27].

#### Force analysis

The MVC force measured from baseline to peak force represented the subjects’ maximal shrug strength. The average submaximal force during a 20% MVC sustained contraction was determined as the average value from five trials. From each trial, the force signal was calculated from the same segment that was used to calculate the RMS EMG activation map.

#### USG outcome

The scapular acetabuli of the patient were pathologically reduced, so the scapulae were in relative protraction and depression. The acromion were also reduced but with normal trapezius muscle insertion. The trapezius insertion areas were in the anatomically correct places, which explained the trapezius muscle’s normal shape.

An ultrasound examination showed that the active part of the trapezius muscle had greater echo intensity in the congenital transverse deficiency subject then in the control subject, which suggests a greater ratio of connective to fat tissue in that muscle [28, 29]. In the patient, thickened skin and subcutaneous tissue overlaying the trapezius muscle under the electrodes grid was on the subject’s left side (10.6 mm) and right side (7.4 mm); in the control subject, the corresponding values were 3.5 mm and 4.0 mm, respectively.

#### Force outcome

The absolute force MVC increased with training (pre- compared to post-12), but the changes were more noticeable in the patient (Table 1).

**Table 1:** Absolute value of submaximal (20%MVC) and maximal (MVC) force [N] during a shrug isometric contraction

	Control		Patient	
	Left side	Right side	Left side	Right side
20% MVC				
Pre	68,23 ± 0,42	64,68 ± 0,33	16,86 ± 0,27	25,70 ± 0,52
Post 4	89,80 ± 0,56	78,28 ± 0,69	14,32 ± 0,17	29,34 ± 0,90
Post 8	82,96 ± 0,72	103,72 ± 0,30	29,98 ± 0,80	35,57 ± 0,36
Post 12	80,10 ± 0,68	71,03 ± 0,52	50,27 ± 1,12	55,96 ± 0,70
MVC				
Pre	314,74 ± 25,05	314,69 ± 17,61	149,67 ± 18,37	181,14 ± 14,64
Post 4	451,50 ± 33,05	393,74 ± 10,75	96,06 ± 19,42	135,11 ± 29,02
Post 8	356,68 ± 4,73	455,65 ± 23,85	143,01 ± 49,15	168,39 ± 7,62
Post 12	310,99 ± 21,90	368,38 ± 4,29	234,64 ± 19,36	271,92 ± 15,59

Values are presented in mean ± standard deviation for each session: before (Pre) and after 4,8 and 12 weeks (Post) of the training

#### HD-EMG repeatability

**Fig. 2:**
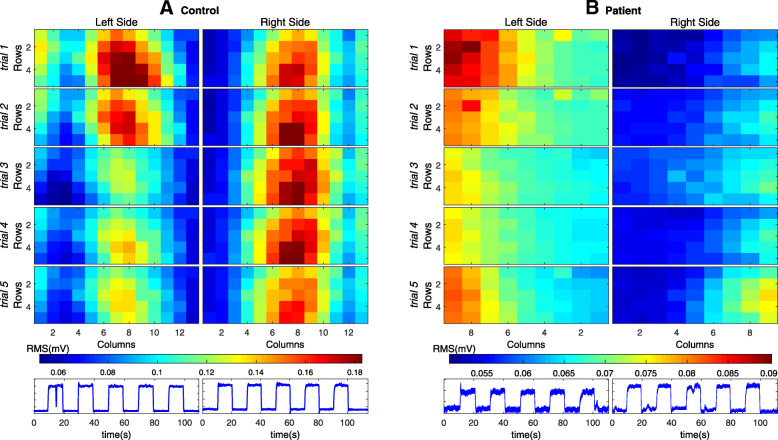
Muscle activation maps *I*_*x,y*_ of the trapezius for five trials after 4 weeks of training. **a** for the control subject; **b** for the bilateral upper limb congenital transverse deficiency patient. The force signal is presented at the bottom

Figure 2a and b show muscle activation maps for the control subject and the patient, respectively. Only the first session after the start of CAT training (that is, in the 4th week) is presented as an example. Maps and force signals for the five trials at the left and right trapezius are presented. We observed that the maps of the different trials had similar spatial distributions and that the force was constantly maintained during the trials. To identify characteristic of the force steadiness output during constant phase force production we used the following formula of the coefficient of variation (CV = (S_t_/ $\overline{x}\Big)$ 100), where s is the square root of the residual variance and $\overline{x}$ is the mean value of the force. The degree of the unsteadiness of the force output was 2.52–7.33% and 1.84–5.24%, between trials of the same sessions respectively for the left and right side fin the patient and 1.43–3.69% and 1.51–4.03% in the control subject.

To assess within subject variability we used the variance (Var) of the average intensity of the maps. Variance is the average squared difference of the values from the mean divided by the number of samples − 1. High values of variance indicate high variability between trials for a specific subject. In addition, we also calculated the cross-correlation (CC) coefficient which is a measure of the similarity between maps. The CC provides a single value of similarity that varies between 0 and 100% (or 0 and 1). Values of the CC are presented in the table in [%] as mean ± standard deviation and in [min, max]. A high Correlation Coefficient close to 100 indicates high similarity between values within the subject (in the session).

We observed that the mean correlation coefficient (CC) was > 90% for both the control subject and the patient and that the lowest values were obtained for the first session, that is, before CAT independent of side. Table 2 shows the correlation coefficient (CC) between trials for different sessions as well as the left and right side.

**Table 2:** Cross–correlation coefficient [%] of activation maps between trials of the same session

	Control		Patient	
	Left Side	Right Side	Left Side	Right Side
Pre	99.15 ± 0.61	93.21 ± 6.8	88.92 ± 8.5	65.27 ± 18
	*[98.22,99.76]*	*[78.82,99]*	*[69.26,96.98]*	*[33.38,91.38]*
Post 4	99.38 ± 0.25	96.61 ± 3.3	95.99 ± 2.6	96.94 ± 1.7
	*[99.01,99.74]*	*[88.14,99.63]*	*[91.2,98.78]*	*[92.74,98.71]*
Post 8	99.38 ± 0.44	99.71 ± 0.2	99.43 ± 0.32	98.94 ± 0.78
	*[98.63,99.88]*	*[99.27,99.92]*	*[98.91,99.89]*	*[97.19,99.87]*
Post 12	99.73 ± 0.12	97.85 ± 1.6	99.6 ± 0.32	99.73 ± 0.18
	*[99.55,99.87]*	*[94.73,99.56]*	*[98.84,99.89]*	*[99.41,99.92]*

Values are presented in mean ± standard deviation and in [*min, max*] for left and right trapezius muscle

for each session: before (Pre) and after 4,8 and 12 weeks (Post) of the training

In addition, Table 3 shows the average variance of intensity between different trials (Var). In all cases, the variance is very low (~ < 0.01 mV). These results show that the spatial distribution and intensity were very similar between different trials of the same session, showing high repeatability.

**Table 3:** Variance of average intensity of the maps [mV] between trials of the same session

	Control		Patient	
	Left side	Right side	Left side	Right side
Pre	0.010	0.002	0.002	0.002
Post 4	0.013	0.005	0.003	0.003
Post 8	0.004	0.012	0.006	0.004
Post 12	0.007	0.005	0.005	0.005

Results are presented for left and right trapezius muscle for each session: before (Pre) and after 4,8 and 12 weeks (Post) of the training

#### Changes in CAT-associated HD-EMG maps

**Fig. 3:**
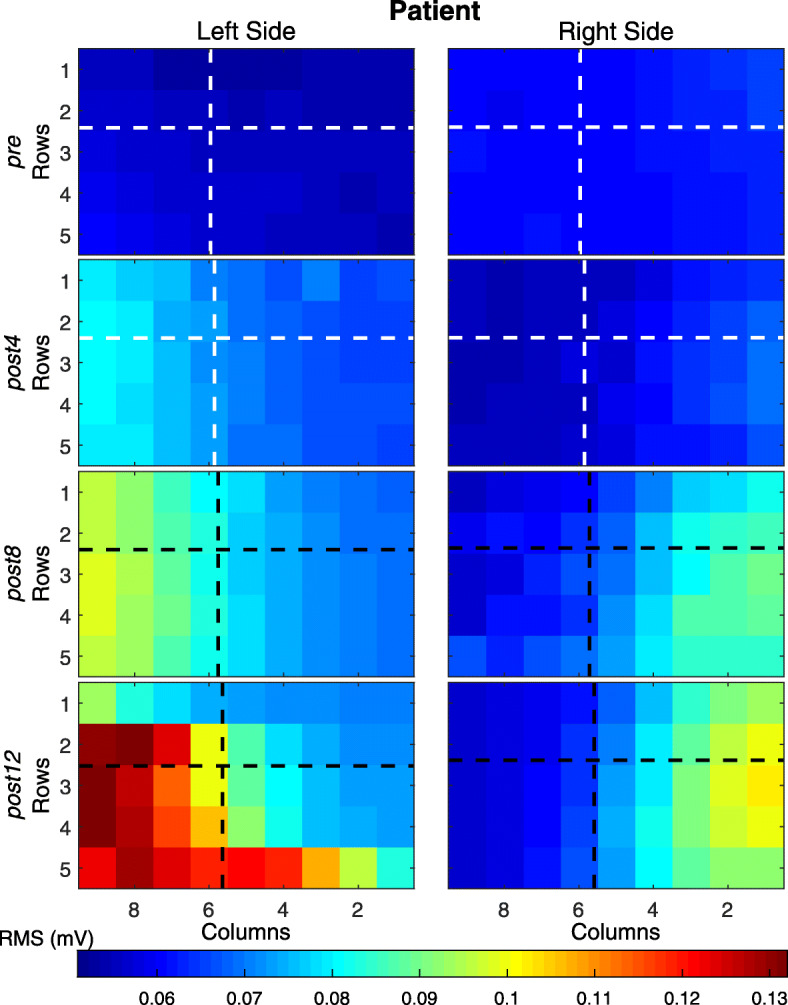
Average muscle activation maps *I*_*x,y*_ for each of the four sessions for bilateral upper limb congenital transverse deficiency patient. pre - before training; post 4,8,12 - after 4, 8, and 12 weeks of the training respectively

Average activation maps for each of the four sessions pre-training and after 4, 8, and 12 weeks are presented in Fig. 3 for the control subject (A) and the patient (B). The coordinates of the *CoG* in each case are presented as continuous lines.

**Fig. 4:**
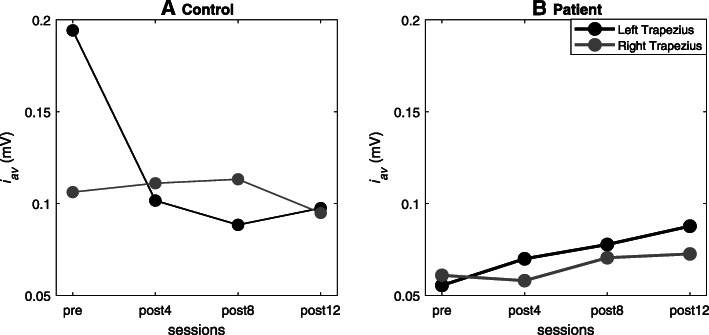
Changes in the average intensity of the electromyographic maps *i*_*av*_ with computer-aided training. **a** for the control subject; **b** for the bilateral upper limb congenital transverse deficiency patient. pre - before training; post4,8,12 - after 4, 8, and 12 weeks of the training respectively

**Fig. 5:**
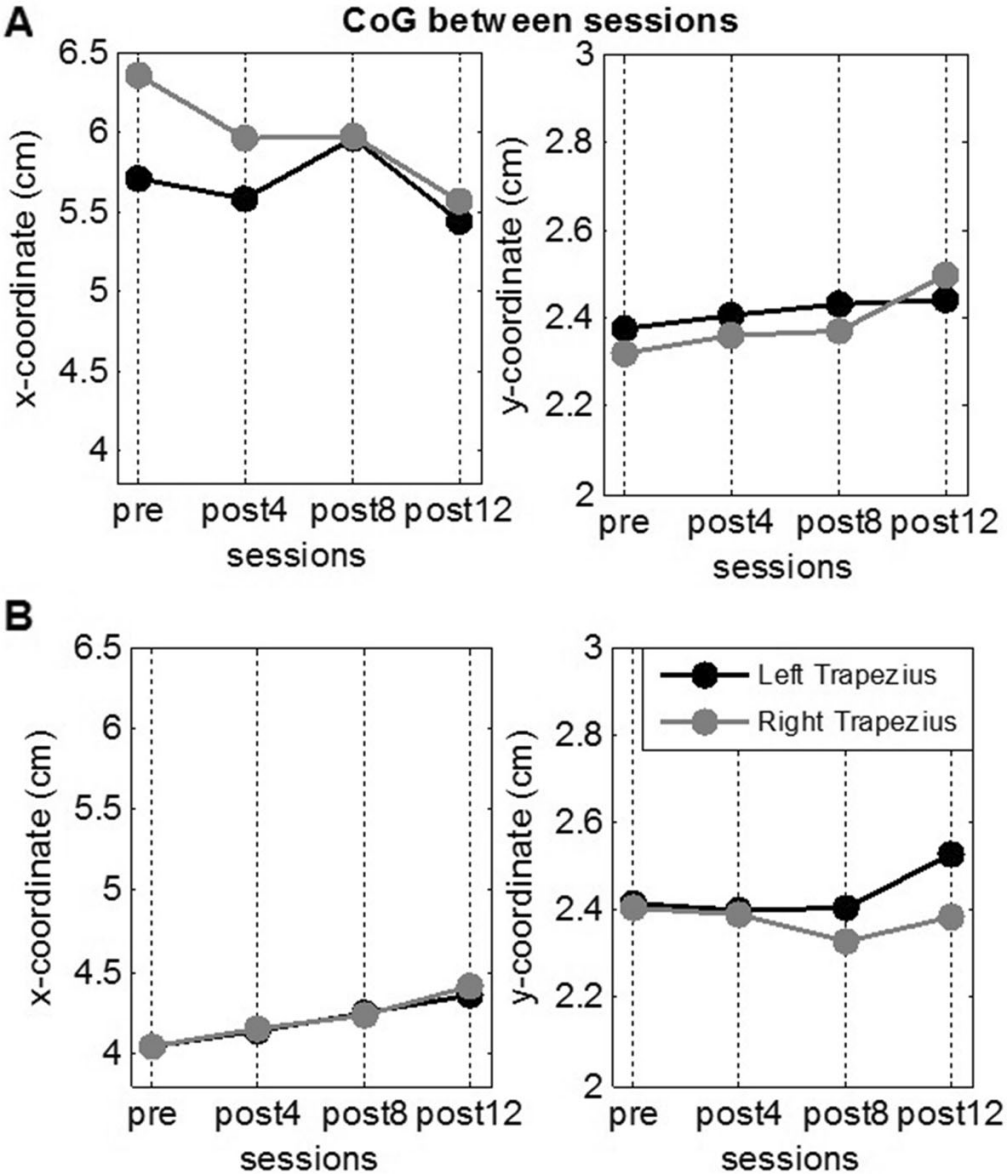
Changes in the center of gravity *(CoG)* on the trapezius muscle with computer-aided training. **a** for the control subject; **b** for the bilateral upper limb congenital transverse deficiency patient. pre - before training; post4,8,12 - after 4, 8, and 12 weeks of the training respectively. The x- and y-coordinates of the *CoG* are presented

We observed that the spatial distribution as well as the average intensity changed between different sessions, especially in the patient. Figures 4 and 5 show the detailed changes in *i*_*av*_ and *CoG,* respectively. We observed that the intensity varied by approximately 50% for the left and right sides between the session before training (*pre*) and after the end of treatment (*post-12*) in the congenital transverse deficiency patient. Similarly, the x-coordinate (left and right) of *CoG* varies almost linearly with time in the congenital transverse deficiency patient, whereas such changes were less uniform in the control subject. Nevertheless, the x-coordinate was displaced by > 4 mm between the first and last sessions, although in opposite directions: from medial to lateral in the control subject and from lateral to medial in the congenital transverse deficiency patient. The y-coordinate had less variation (~ 2 mm) in the same direction from the cranial to caudal end for the control subject and the congenital transverse deficiency patient (for the left and right trapezius).

Compared to our patient, changes in the average intensity of the electromyographic maps with computer-aided training (between sessions) was less uniform for the left and right sides in the control subject (with link to changes of the force generation). We observed that the intensity was more varied for the left side as compared to right side between the sessions in the control subject. More diverse values of the intensity in the control subject (between sessions and between trials of the same sessions) could be associated with the nature of the control strategy of the multiple muscles (the synergist and antagonist muscles) to generate the force output in different trials. Several studies have observed differences in the control strategies of right and left upper trapezius pointing to an asymmetrical activation of the trapezius that is associated with side dominance (see for example [30, 31]). Therefore, the observed differences in Fig. 4 can be attributed to the dominance of the control subject (who in this case is right-handed). Furthermore, the trapezius muscle is not a prime mover in reach movement (training motor task) however, because of lack of upper limbs in our patient, we had to choose a muscle that is at least in some extent active in both shrug and reach. This can partly explain less uniform for the left and right sides changes in the trapezius activation due to the computer-added training in the control subject.

#### Innervation zone (IZ) localization of the horizontal middle division of the trapezius muscle

**Fig. 6:**
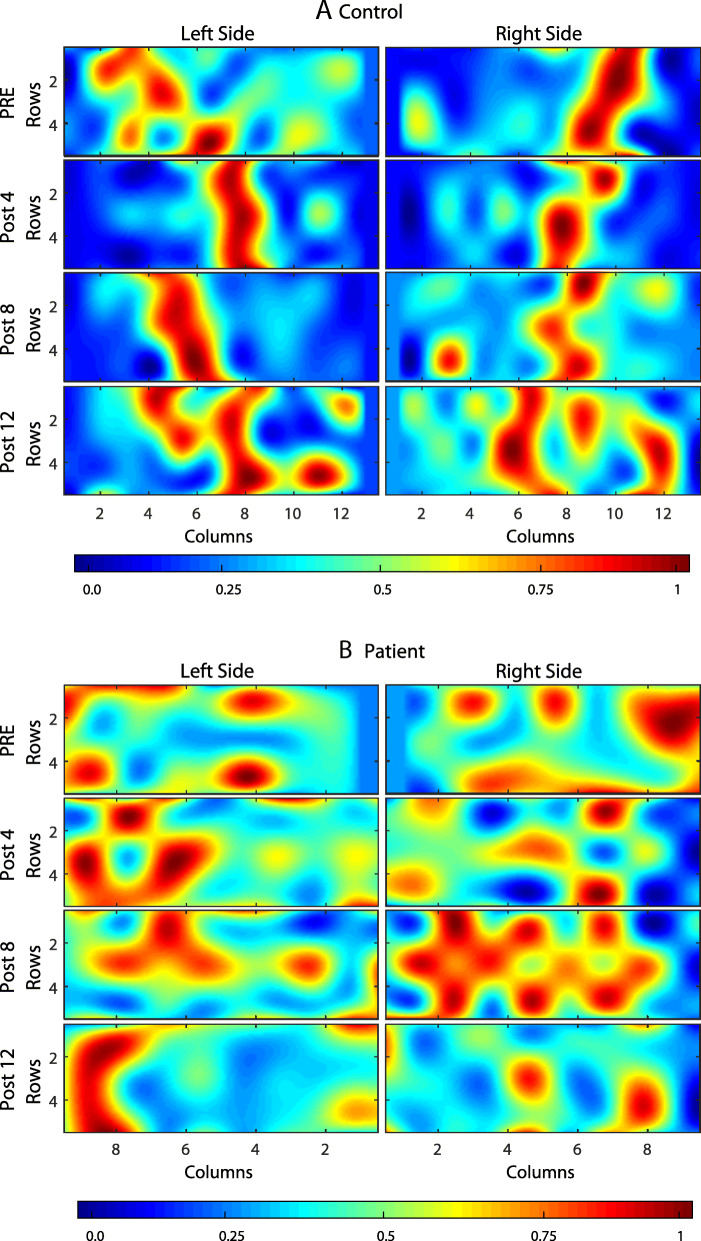
Innervation zone maps of the horizontal middle division of the trapezius muscle during the shrug isometric contractions. **a** for the control subject; **b** for the bilateral upper limb congenital transverse deficiency patient. pre - before training; post4,8,12 - after 4, 8, and 12 weeks of the training respectively. The maximum cross-correlation coefficient was normalized to 0 (dark blue) and + 1 (dark red, localized IZ)

In the present study, the IZ of the horizontal middle division of the trapezius muscle were clearly observed in the healthy subject (Fig. 6). The IZ from pre- to after 8 weeks of training were distributed in a 16-mm band around the middle part of the muscle with a slight slope from the medial to lateral direction for the right and left sides. After 12 weeks of training, the IZ were distributed in a wide area ranging from the 3rd to the 10th columns for the left side and from the 5th to the 12th columns for the right side.

In the patient with bilateral upper-limb congenital transverse deficiency, the IZ of the trapezius muscle region could not be not clearly detected. They were located irregularly on the entire map before training and after 8 weeks of training (Fig. 6). After 12 weeks of training, the IZ were distributed throughout a narrow area (20 mm) ranging from the 7th to the 9th columns for the left side and in two regions of the muscle (from the 4th to the 5th and from the 7th to the 8th columns) for the right side of the body.

## Discussion and conclusions

The novelty of our study is that we trained a bilateral congenital transverse deficiency patient to perform a reaching to grasp (RTG) movement using CAT and recorded HD-EMG maps of the trapezius muscle. We observed some differences in changes in the spatial distribution, and the average intensity between different training sessions during the 12-week intervention period.

In everyday life, our bilateral upper-limb congenital transverse deficiency patient uses her shoulders to shore up and hold objects (e.g., cellular phone) but her lower extremities to reach for objects. Thus, in line with the motor program theory, our patient performed a reach and grasp task, although she has always executed the movement using her lower extremities (in contrast to our control). In addition, because of the lacking upper limbs, the trapezius muscle of the patient never had to counteract the limb’s weight or be involved in reaching movement initiation or hand transition. During CAT, relying only on vision, our patient had to learn to use her upper extremities to reach, grasp, and return to the starting position following virtual upper-limb movements because the only muscles that she has and can use to execute the reach task are the muscles adjacent to the undeveloped limb. Thus, we chose to study the trapezius muscle fibers with transverse orientation (medial trapezius) as their action is to draw the clavicle, acromion, and scapula backward and medially [22], movements that contribute to transitioning the upper limb from the grasp back to the starting position as well as performing the shrug motion.

### CAT increases muscle activity and strength in congenital transverse deficiency subject

By analyzing learning of the reaching task during training, we observed a large bilateral increase in shrug force (more than double) in our congenital transverse deficiency patient. This was a result of increased motor unit activity mirrored in the increased bilateral intensity of the activation maps in our patient accompanied by variation of the left and right x-coordinates of the *CoG*. The economization process of motor unit activation during learning and the existence of functionally independent muscle segments (motor units from within a muscle that are simultaneously activated for a particular motor task, e.g. reach) [32] may explain these findings.

Compared to our patient, the influence of training in the healthy individual was smaller and less regular. Displacement of the x-coordinate between the first and last training sessions occurred in opposite directions in the healthy subject versus the patient: from the medial to lateral side in the control subject and from the lateral to medial side in the congenital transverse deficiency subject. The difference can be related to the lack of proprioceptive information in the patient and thus different control of functional muscle deviations [33]. Monaco et al. [34] showed that reaches rely more heavily on proprioceptive information than saccades and that proprioception is of greater relative utility for determining reach amplitude, whereas vision is required for direction of reach. Sober and Sabes [35] also found that the position estimate used for movement vector planning relies mostly on visual input, whereas the estimate used to compute the joint-based motor command relies more on proprioceptive signals. Thus, as our patient lacked proprioceptive information from the upper limb, she had to rely on vision alone for determining reach amplitude and direction (using a reference frame outside the body), whereas the control subject utilized both proprioception for determining reach amplitude (using a reference frame based on the body such as joint angles) and vision for direction (using a reference frame outside the body). Moreover, in contrast to the patient, the healthy control used her trapezius muscles in everyday activities as well as for initiation reaching and bearing limb weight. These differences can induce different physiological factors such as fiber type and contractile element volume; thus, this explains the existence of different motor unit task groups in the trapezius muscle of our control and patient participants.

The results, although derived from only one congenital transverse deficiency subject, suggest that CAT training of the RTG task could systematically increase the mean EMG activity within the region of the trapezius muscle and improve muscle strength of the shrug movement from the 4th to 12th weeks of training. These observations extend the findings of previous studies showing that kinesthetic imagery training without physical exercise can increase the motor-related cortical potential of the sensorimotor cortex amplitude, EMG signal amplitude, and muscle force [36, 37] by increasing the voluntary neural drive to the muscle [38]. Our present study showed that individuals born without both upper extremities could successfully perform mental training of the reaching to grasp of the virtual limb. During reaching and grasping of the book with four fingers and the thumb, the trapezius muscle is not a prime mover and its EMG activity is relatively small. However, our results demonstrated that the mental training of the RTG task can change muscle activity of the trapezius muscle, the shoulder muscle used during the isometric motor task (shrug). These results showed the potential benefits of mental training in the rehabilitation process by transferring the motor behavior.

### Scattered IZ localization in congenital transverse deficiency subject changed after training

In the present study, the distribution of IZ of the horizontal middle division of the trapezius muscle was clarified in the bilateral upper-limb congenital transverse deficiency patient and the healthy subject. The neuromuscular junction or end-plate where the depolarization begins, can be estimated by using multichannel surface EMG. Interpretation of the distribution of innervation zones depends on the simplicity in detecting the propagation of motor units action potentials (MUAPs) which is linked with muscle morphology with respect to the arrangements of muscle fibers. In the healthy subject, the IZ of the region of the trapezius muscle were located at the middle length of the map in a rather narrow band with a slight slope from the medial to lateral direction on both sides (left and right) before and after training. Our results in the congenital transverse deficiency patient demonstrated that the IZ of the of trapezius muscle were not distributed in the middle length of the muscle but irregularly on the whole map before and after training. This could be associated with different muscle architectures (fiber length and diameter, the “End of Fiber” effect), amount of connective tissue (on the USG scan), and motor endplates spread throughout the muscle, number of motor units, innervation index of the motor units and different conduction velocity. These are possible reasons as to why the IZ of the trapezius muscle were scattered widely throughout the trapezius muscle in the congenital transverse deficiency subject. The scattered IZ of the trapezius muscle in the congenital transverse deficiency subject became visible and more focused after the 12-week training program. These results show that 12 weeks of the CAT may induce the changes related within the origin (source) of the MUAPs of the activated motor units. We speculated that such reorganization may be due to the “fine tune” motor unit activation, recruitment of new motor units, and/or coordination of motor unit activity timing and intensity within the trapezius muscle after training. The detection of the changes after training in the congenital transverse deficiency subject could be associated with specific modification of the neural drive to the muscle (from the central nervous system – the motor command) and the peripheral features of the muscle. These results are supported by the increased force level noted after training.

In summary the CAT-induced changes in trapezius activation as well as IZ changes in our patient compared to our control subject indicated that the CNS adjusts the activity of independent trapezius muscle functional segments by “fine tuning” trapezius motor unit activity during the control of reach and shrug movements and can do so through its upper and lower motoneurons.

Here we reported differences in an individual with bilateral upper-limb congenital transverse deficiency and a healthy control in muscle activation maps of the trapezius muscles because of CAT of the reach to grasp movement with a larger left-to-right body side value in the control subject and different changes in spatial distribution and mean intensity between different CAT sessions in the patient. Our data shows that individual with bilateral upper-limb congenital transverse deficiency are capable to imagine reaching and grasping a book with the computer-generated hand without movements (monitored by EMG). The motor imagery based - CAT may be beneficial in congenitally amputated patients before upper-extremity transplantation to induce plasticity of the neuromuscular system.

## Data Availability

The datasets generated and/or analysed during the current study are not publicly available due to ethical restrictions but are available from the corresponding author on reasonable request.
